# Evaluation of pulse-oximetry oxygen saturation taken through skin protective covering

**DOI:** 10.1186/1471-2431-6-14

**Published:** 2006-05-06

**Authors:** Jyotsna James, Lokesh Tiwari, Pramod Upadhyay, Vishnubhatla Sreenivas, Vikas Bhambhani, Jacob M Puliyel

**Affiliations:** 1 St Stephen's Hospital Tis Hazari, Delhi 110054, India; 2 Centre for Science Education and Communication, University of Delhi, India; 3 Department of Bio-statistics All India Institute of Medical Sciences, New Delhi, India

## Abstract

**Background:**

The hard edges of adult finger clip probes of the pulse oximetry oxygen saturation (POOS) monitor can cause skin damage if used for prolonged periods in a neonate. Covering the skin under the probe with Micropore surgical tape or a gauze piece might prevent such injury. The study was done to see if the protective covering would affect the accuracy of the readings.

**Methods:**

POOS was studied in 50 full-term neonates in the first week of life. After obtaining consent from their parents the neonates had POOS readings taken directly (standard technique) and through the protective covering. Bland-Altman plots were used to compare the new method with the standard technique. A test of repeatability for each method was also performed.

**Results:**

The Bland-Altman plots suggest that there is no significant loss of accuracy when readings are taken through the protective covering. The mean difference was 0.06 (SD of 1.39) and 0.04 (SD 1.3) with Micropore and gauze respectively compared to the standard method. The mean difference was 0.22 (SD 0.23) on testing repeatability with the standard method.

**Conclusion:**

Interposing Micropore or gauze does not significantly affect the accuracy of the POOS reading. The difference between the standard method and the new method was less than the difference seen on testing repeatability of the standard method.

## Background

**Figure 1:**
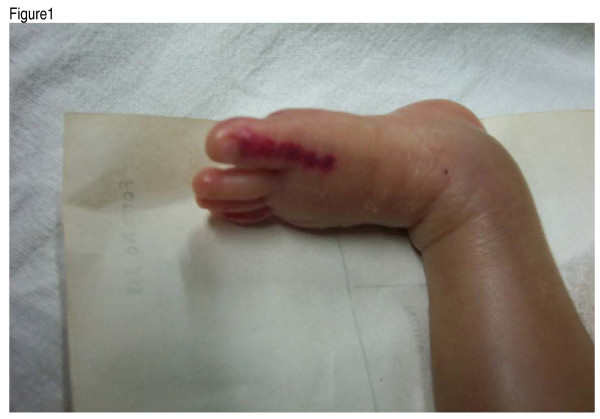
Showing pressure injury to the foot of a neonate through application of a adult clip probe for saturation monitoring

Pulse-oximetry oxygen saturation (POOS) monitors are now commonplace in paediatric intensive care areas and in neonatal units. A variety of probes are available for use with saturation monitors. Adult finger-clips have a longer useful life than neonatal clip probes and disposable wrap-around probes. In the long run, adult finger clips are cheaper to use and we have been using them across the neonate's palm or feet for obtaining saturation readings. Sensor placement over the palm or foot of the neonate has been described previously [1]. However the rigid edges of these probes when used for long periods can cause injury and skin necrosis as shown in figure 1. Injuries may be prevented if the palm or sole is first covered with a layer of 3 M Micropore surgical tape or 2 layers of gauze. This study was done to test the degree of inaccuracy introduced in the POOS by the use of Micropore or gauze in this manner.

## Methods

A convenience sample of fifty neonates was enrolled in the study after obtaining verbal consent from their parents. The neonates were all born in the hospital, less than a week old, and admitted on the post-natal ward. No effort was made to select babies – so as to get a wide range of readings. Babies with low saturations were treated as per hospital protocol. Each neonate had readings taken thrice, at intervals of 5 minutes, using a Larsen & Tourbo Stellar pulse oximeter (Hebbal, Mysore India). The POOS monitor readings were taken in random order with the right foot covered with 3 M Micropore surgical tape (1530-1 hypoallergenic. St Paul USA) or covered with two layers of gauze (weight 27 gm/sq m +/-5%: Government of India specification) or directly without any covering. Readings were noted after the saturation display had steadied. The investigator noting the readings was seated so that he could read the saturation monitor but he was blinded as to how the probe was applied. The readings with Micropore and gauze were each compared with the readings taken with the probe applied directly (standard method). Five minutes later the procedure was repeated on the opposite limb. Only the first measurements were used to illustrate comparison of methods. The second measurement was used in the study of repeatability – a technique described by Bland and Altman [2].

### Statistical methods

A 0.05 two-sided Fisher's z test of the null hypothesis suggests that the Pearson correlation coefficient *r *= 0.50 will have 90% power to detect an *r *of 0.0 when the sample size is 38. We used a sample size of 50 for greater confidence in the findings.

Bland-Altman plots were used to study agreement between methods and to test the repeatability of the methods [2]. As a first step, the data was plotted and the line of equality was drawn (a line on which all points would lie if the two methods gave exactly the same reading every time). Then a plot of the difference between methods against their mean was made. The estimated mean difference d and standard deviation s of the difference were calculated. We would expect most of the differences to lie between d -2s and d +2s. Bland and Altman suggest that provided the differences within d +/- 2s are not clinically important, we could use the two measurements interchangeably.

## Results

**Figure 2:**
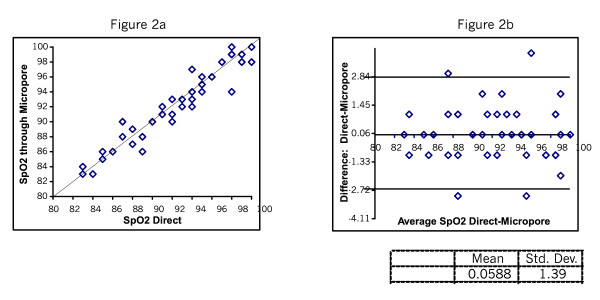
SpO_2_ Direct and through Micropore

**Figure 3:**
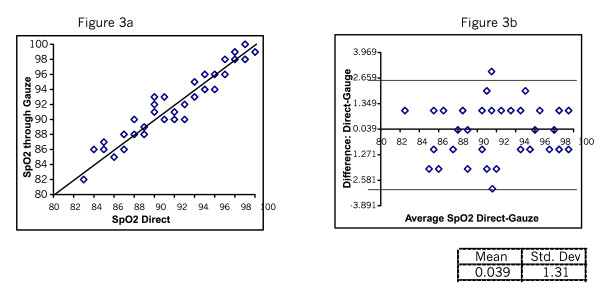
SpO_2_  Direct and through Gauze

**Figure 4:**
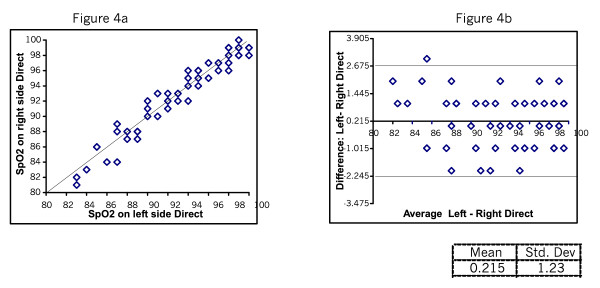
Reapeated meausures of SpO_2_ with direct method

**Figure 5:**
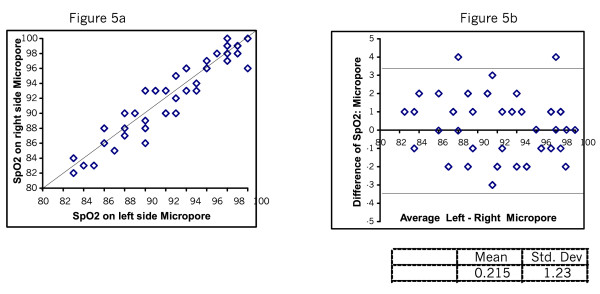
Repeated measures of SpO_2_ through Micropore

**Figure 6:**
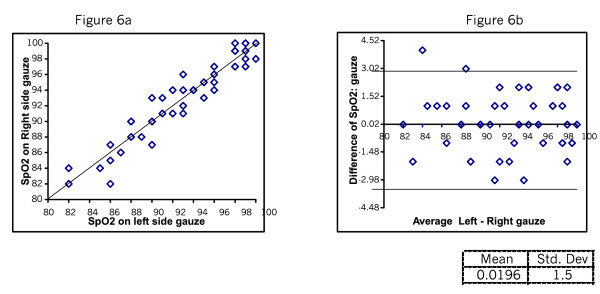
Repeated measures of SpO_2_ through Gauze

Figure 2a and 2b shows the SpO_2 _readings taken directly and through Micropore. The mean difference was 0.06 with SD of 1.39. Figure 3 (a & b) shows the SpO_2 _readings taken directly and through gauze. The mean difference was 0.04 SD 1.3. Figure 4 (a & b) shows the plots testing repeatability with the direct method. The mean difference was 0.22 SD 0.23. Figure 5 (a & b) shows mean difference on repeatability of readings through Micropore. The mean difference was 0.23 SD 1.69. Figure 6 (a & b) shows repeatability of readings through gauze. The mean difference was 0.02 SD 1.50.

## Discussion

Theoretically, if the translucent material (interposed between the light emitting diode and the photo detector sensor in a POOS) is more transparent to one of the two wavelengths used by the POOS diode, it will induce an error and the machine will give a false reading. We employed the Bland-Altman plots to look at the comparability of methods. It is seen that difference between readings taken through the skin protecting coverings (Micropore and gauze) and the readings taken directly are not very different from the differences seen on testing repeatability of the direct method. These differences can therefore be considered clinically insignificant. Our findings suggest that interposing Micropore or gauze does not affect the accuracy of the readings to a clinically significant level in the range of saturations we studied.

Bland and Altman have compared the oxygen saturation meter and pulsed saturation oxymeter. They found a mean difference of 0.42 percentage points with 95% CI 0.13 to 0.70. They concluded that the limits of agreement (-2.0 and 2.8) were small enough for confidence that the new method can be used in place of the old, for clinical purposes^22)^. Hess and colleagues have found that the standard error on using the probe directly was 2% (95% confidence interval was about +/-4%) [3]. Alexander et al found that the 95% prediction limit of a single pulse oximeter reading was +/- 6% throughout the 70–100% range of saturation, such that there is a 95% probability that an oximeter reading of 90% corresponds to an arterial saturation between 84 and 96% [4]. We found that the agreement between the direct method and readings taken through Micropore (and through gauze) was less than the variations taken as acceptable by previous authors.

We also found on examining repeatability of the direct method that the mean difference was 0.22 with SD of 1.23.

This test of repeatability of the direct method defines what must be considered acceptable because such variability is inherent in using the standard method. The differences we found between methods (and the differences that were shown on repeatability testing through Micropore and through gauze) were less or only marginally more than the variability seen on testing reproducibility of the direct method. This is a novel use of the Bland-Altman test of repeatability of the standard method to define the range of what must clearly be "acceptable differences" between methods.

We have not used blood gas readings as the gold standard for comparison. This was not considered necessary as Bland-Altman plots were employed. According to Bland and Altman, a 'gold standard' is needed for comparison only for calibration. [2] They suggest that "new methods" can be compared to an "established technique" rather than with the true quantity. If the new method agrees sufficiently well with the old the old may be replaced.

The practice of using adult probes in neonates is not confined to resource scarce countries. Recent Advances in Pediatrics 18 describes how to use adult probes if infant probes are not readily available [5]. In India, adult clip probes (Oximax Durasensor DS 100A Nellcor USA) cost US $175 and lasts for 6 – 9 months in our nursery. The neonatal clip probe (Dura Y sensor Nellcor USA) costs US $188 and lasts for a month. The disposable neonatal sensor costs about US $16 and is for single use only.

## Conclusion

In conclusion, considerable saving can be achieved with the reusable adult probe by using 3 M Micropore or a gauze piece to wrap around the skin below the clip, and this practice does not compromise accuracy of readings taken by POOS monitors.

## Authors' contributions

JJ, VB, LT and JMP devised the study. JJ, VB and LT did the clinical trial and PU helped with the physics. VS did the statistical analysis. JJ, LT, PU, and JMP wrote the paper

## Pre-publication history

The pre-publication history for this paper can be accessed here:


